# Activity of pemetrexed in pre-clinical chordoma models and humans

**DOI:** 10.1038/s41598-023-34404-4

**Published:** 2023-05-05

**Authors:** Santosh Kesari, Feng Wang, Tiffany Juarez, Shashaanka Ashili, C. Pawan K. Patro, Jose Carrillo, Minhdan Nguyen, Judy Truong, Joan Levy, Josh Sommer, Daniel M. Freed, Joanne Xiu, Yuki Takasumi, Eric Bouffet, Jaya M. Gill

**Affiliations:** 1 Department of Translational Neurosciences, Pacific Neuroscience Institute, Santa Monica, CA USA; 2 Saint John’s Cancer Institute at Providence Saint John’s Health Center, Santa Monica, CA USA; 3 Department of Medical Oncology, Cancer Center, West China Hospital, West China Medical School, Sichuan University, Sichuan, Chengdu China; 4 CureScience, San Diego, CA USA; 5 Cancer Science Institute of Singapore, National University of Singapore, Singapore, Singapore; 6 Chordoma Foundation, Durham, NC USA; 7 CARIS Life Sciences, Phoenix, AZ USA; 8 The Hospital for Sick Children (SickKids), University of Toronto, Toronto, Canada

**Keywords:** Cancer therapy, Sarcoma, Chemotherapy

## Abstract

Chordomas are rare slow growing tumors, arising from embryonic remnants of notochord with a close predilection for the axial skeleton. Recurrence is common and no effective standard medical therapy exists. Thymidylate synthase (TS), an intracellular enzyme, is a key rate-limiting enzyme of DNA biosynthesis and repair which is primarily active in proliferating and metabolically active cells. Eighty-four percent of chordoma samples had loss of TS expression which may predict response to anti-folates. Pemetrexed suppresses tumor growth by inhibiting enzymes involved in folate metabolism, resulting in decreased availability of thymidine which is necessary for DNA synthesis. Pemetrexed inhibited growth in a preclinical mouse xenograft model of human chordoma. We report three cases of metastatic chordoma that had been heavily treated previously with a variety of standard therapies with poor response. In two cases, pemetrexed was added and objective responses were observed on imaging with one patient on continuous treatment for > 2 years with continued shrinkage. One case demonstrated tumor growth after treatment with pemetrexed. The two cases which had a favorable response had a loss of TS expression, whereas the one case with progressive disease had TS present. These results demonstrate the activity of pemetrexed in recurrent chordoma and warrant a prospective clinical trial which is ongoing (NCT03955042).

## Introduction

Chordomas are rare slow-growing tumors that arise from embryonic remnants of notochord and have a close predilection for the axial skeleton including the cranium, spine, and sacrum^1,2^. Chordomas are an extremely rare cancer with an incidence of one case per million persons in the US^1^. Although chordomas are considered low-grade neoplasms, they are highly recurrent, making their clinical progression similar to malignant tumors^3^. The median overall survival from the time of diagnosis is 6–7 years, with the 5- and 10-year relative survival rates being 67.6% and 39.9%, respectively^1^.

Currently, the mainstay of treatment for newly diagnosed chordoma is maximum surgery which has a good success rate, followed by radiotherapy, most often proton^4,5^. Recurrent chordomas are usually treated with surgery when feasible^6,7^. Unfortunately, wide local excision is not always an option for patients. In addition, the histology of the tumor has shown that despite careful planning, gelatinous material can spill into the resection cavity, and recurrence often occurs at the margins of the resected tumor. Therefore, patients often receive high-dose radiotherapy after full or subtotal resection and also as a primary treatment in those with unresectable disease^8^. In recurrent disease, radiotherapy after surgery has poor outcomes^9^. Approximately 30% of chordomas eventually metastasize, predominately to the skin, bone and brain^10^. However, most patients die of local–regional disease even when metastases occur^11^. To date, no standard systemic treatment is approved, leaving a high unmet need for effective medical therapy.

Pemetrexed is a newer anti-folate that antagonizes nucleic acid synthesis by inhibiting dihydrofolate reductase (DHFR), thereby disrupting folate-dependent metabolic processes essential for cell replication. Unlike the anti-folate methotrexate, pemetrexed also inhibits thymidylate synthase (TS) and glycinamide ribonucleotide formyl transferase (GRAFT), and to a lesser extent aminoimidazo carboxamide formyltransferase (AICARFT) and C1-tetrahydrofolate synthase^12,13^. Pemetrexed gains entry to the cell via the reduced folate carrier and once localized, is an excellent substrate for folypolyglutamate synthase (FPGS), with the highest affinity of any antifolate. Pemetrexed is transported intracellularly where it is metabolized to its predominant pentaglutamate and found to be at least 60-fold more potent in its inhibition of TS than the monoglutamate^12^. Additionally, pemetrexed is 90- to 195-fold more efficiently polyglutamated than methotrexate^14^. By targeting such enzymes in the folic acid pathway, pemetrexed can be seen as having a broader spectrum of clinical activities than methotrexate and may preclude the development of drug resistance by alterations to a single enzyme^15^.

The ability of pemetrexed to inhibit thymidylate synthase (TS) is particularly important because it is an intracellular enzyme that provides the sole de novo source of thymidylate, making it a key rate-limiting enzyme of DNA biosynthesis and repair. TS is primarily active in proliferating and metabolically active cells. The primary mechanism of action of pemetrexed is inhibition of TS^16^, resulting in decreased availability of thymidine which is necessary for DNA synthesis. Preclinical studies in colon, breast, and non-small cell lung cancer (NSCLC) cell lines demonstrated that high TS expression confer a reduced sensitivity to pemetrexed^17,18^. Xenograft studies in mice have shown that tumors with TS overexpression are resistant to growth inhibition by pemetrexed compared to controls^17,19,20^. These results reveal an inverse correlation between tumor response to pemetrexed and TS expression and suggest that TS levels may be a predictive biomarker for pemetrexed response in cancers including chordoma^21^.

In efforts to identify potential therapeutic targets for refractory chordoma, we characterized the biomarker profile (including TS) of 68 chordoma cases by immunohistochemistry (IHC)^22^. Here we report data using human chordoma xenograft models in in vivo mouse models and the clinical response from three patients with metastatic chordoma that failed multiple treatments and were treated with high-dose pemetrexed.

## Results

### TS expression and molecular profiling in chordoma

We interrogated the CARIS Molecular Intelligence® database and identified 68 cases of chordoma and review of immunohistochemistry (IHC) biomarker results (Table 1) revealed eight-four percent of these chordoma samples had loss of TS expression suggesting that these tumors may respond to anti-folate drugs.

**Table 1:** Immunohistochemistry tumor expression from CARIS database of sixty-eight chordoma cases.

IHC tumor expression	Positive	Total	Percent (%)
MGMT	37	46	80
EGFR	18	24	75
ERCC1	26	39	67
TLE3	15	25	60
PTEN	26	45	58
PGP	22	40	55
TOPO1	28	52	54
SPARC monoclonal	8	20	40
PD-1	8	23	35
TUBB3	14	42	33
PD-L1 (SP142)	8	34	24
TOP2A	10	57	18
TS	8	50	16
PDGFR	2	14	14
RRM1	6	48	13
cMET	2	24	8
SPARC polyclonal	1	24	4
ER	1	43	2
ALK	0	9	0
c-kit	0	17	0
Her2/Neu	0	48	0
PR	0	43	0
TrkA/B/C	0	7	0

*MGMT* O^6^-methylguanine (O^6^-MeG)-DNA methyltransferase, *EGFR* Epidermal Growth Factor Receptor, *ERCC1* Excision Repair Cross-Complementation Group 1, *TLE3* Transducin-like enhancer of split 3, *PTEN* Phosphatase Tensin Homologue, *PGP* proline-glycine-proline, *TOPO1* Topoisomerase 1, *SPARC* Secreted Protein Acidic and Rich in Cysteine, *PD-1* Programmed cell death protein 1, *TUBB3* Tubulin Beta 3 Class III, *PD-L1* Programmed death-1 ligand 1, *TOP2A* Topoisomerase II alpha, *TS* Thymidylate Synthase, *PDGFR* Platelet-derived growth factor receptor, *RRM1* ribonucleotide reductase M1, *cMET* tyrosine-protein kinase Met, *ER* Estrogen receptor, *ALK* anaplastic lymphoma kinase, *c-kit* tyrosine-protein kinase, *Her2/Neu* human epidermal growth factor receptor-2, *PR* Progesterone receptor, *Trk* tropomyosin receptor kinase.

**Figure 1:**
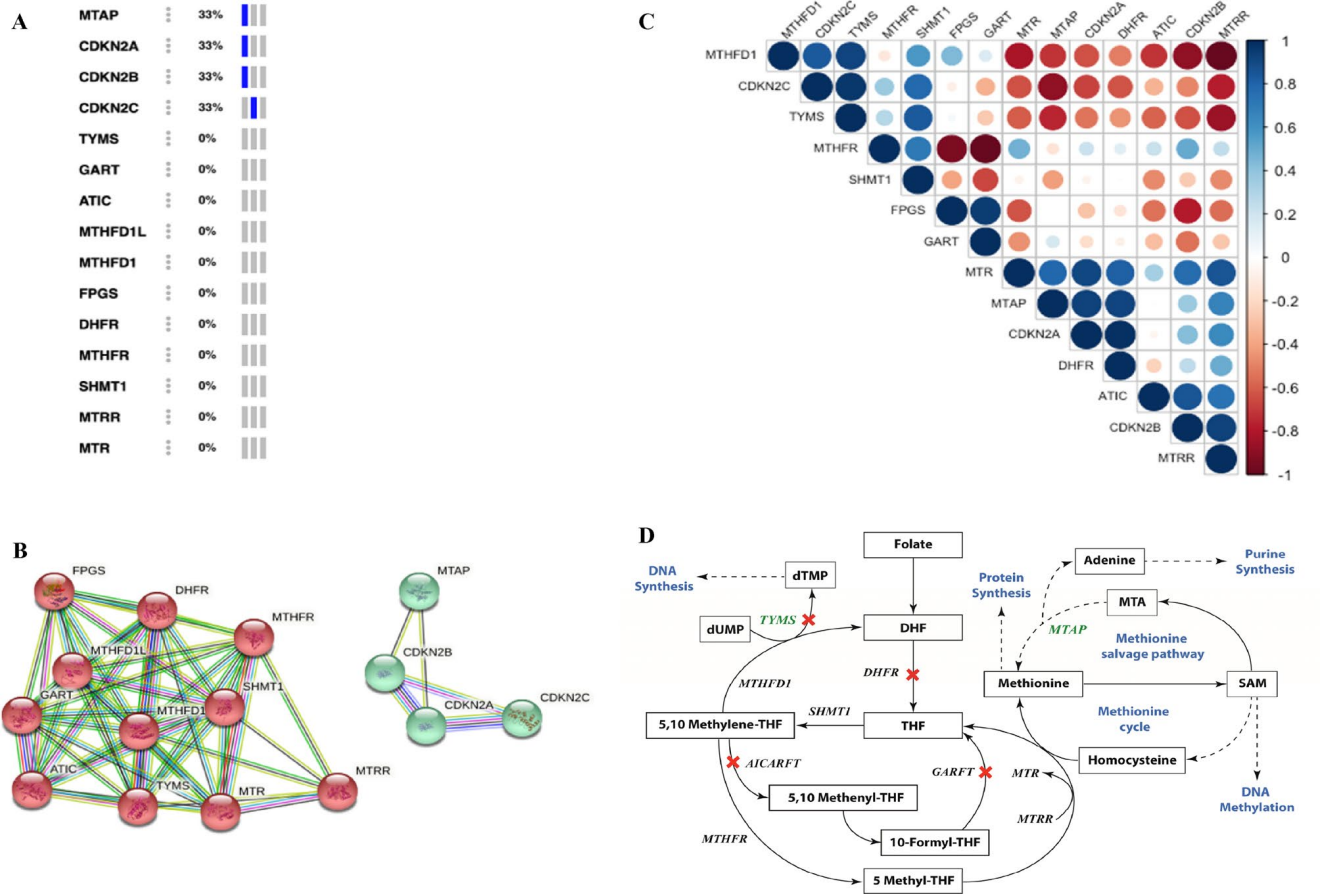
Folate pathway genes and pemetrexed targets. (**A**) Genetic alteration (deep deletion) of specific genes in Chordoma accessed using cBioPortal. Out of the three samples, samples with deep deletion are shown in blue and samples without any deep deletion are shown in grey (**B**). Protein–protein interaction network obtained using STRING database. Edges represent protein–protein associations—blue and pink lines show known interactions from curated databases and experimentally determined interactions respectively; green (gene neighborhood), red (gene fusions) and blue (gene co-occurrence) lines show predicted interactions; light green lines denote text mining, black lines denote co-expression and light purple lines denote protein homology. (**C**). Expression correlation analysis of Chordoma samples obtained from Array Express dataset E-MEXP-353. Correlation values range from 1 to − 1. Positive correlations are shown in shades of blue and negative correlations are shown in shades of red depending on the correlation value. (**D**). Folate metabolism pathway and its link to MTAP. Folate gets metabolized to form DHF and then THF by DHFR. THF gets converted to 5,10-Methylene THF and then 5-Methyl THF. 5-Methyl THF gets converted back to THF by MTR. In a parallel process homocysteine gets converted to methionine. Methionine is at the core of methionine salvage pathway and the methionine cycle. MTAP metabolizes MTA through the methionine salvage pathway producing methionine and adenine required for protein synthesis and purine synthesis respectively. Methionine gets converted to SAM and homocysteine through a series of steps in the methionine cycle. TYMS is involved in conversion of dUMP to dTMP leading to DNA synthesis process. Targets of pemetrexed are marked by a red cross. The key genes TYMS and MTAP are highlighted in green. Cellular processes and metabolism pathways are shown in blue. Dashed arrows represent series of intermediate steps. *MTAP* methylthioadenosine phosphorylase; *CDKN2A* cyclin dependent kinase inhibitor 2A; *CDKN2B* cyclin dependent kinase inhibitor 2B; *CDKN2C* cyclin dependent kinase inhibitor 2C; *TYMS* thymidylate synthetase; *FPGS* folylpolyglutamate synthase; *DHF* dihydrofolate; *THF* tetrahydrofolate; *ATIC* 5-aminoimidazole-4-carboxamide ribonucleotide formyltransferase/IMP cyclohydrolase; *GART* phosphoribosylglycinamide formyltransferase, phosphoribosylglycinamide synthetase, phosphoribosylaminoimidazole synthetase; *MTHFD1* methylenetetrahydrofolate dehydrogenase, cyclohydrolase and formyltetrahydrofolate synthetase 1; *DHFR* dihydrofolate reductase; *MTHFR* methylenetetrahydrofolate reductase; *SHMT1* serine hydroxymethyltransferase 1; *MTRR* 5-methyltetrahydrofolate-homocysteine methyltransferase reductase; *MTR* 5-methyltetrahydrofolate-homocysteine methyltransferase; *SAM* S-adenosyl methionine; *MTA* Methylthioadenosine.

We analyzed the genes in the folate pathway and the targets of pemetrexed^15,23–28^. The CDKN2 genes are located next to MTAP gene on chromosome 9 and thus co-deleted in cases in most cases of CDKN2 loss. The genetic alteration (deep deletion) frequency showed that 1/3rd of the chordoma samples have MTAP loss and CDKN2A, CDKN2B and CDKN2C loss; however, there is no loss in the folate pathway genes in the samples analyzed in personalized oncogenomics cBioPortal (Fig. 1A). The protein–protein interaction network obtained using STRING database showed that the folate pathway genes formed one cluster, and the MTAP, CDKN2A, CDKN2B and CDKN2C formed another cluster with robust interactions; co-expression was observed among some of the genes (Fig. 1B). The expression correlation analysis of the Array Express dataset E-MEXP-353 showed that MTAP, CDKN2A, CDKN2B and CDKN2C expression correlated with few of the folate pathway genes, and high levels of positive and negative correlation are observed among some of the genes (Fig. 1C). Expression of Dihydrofolate reductase (DHFR), which is the key enzyme in folate metabolism, is highly correlated with MTAP and CDKN2A. Expression of Methionine synthase (MTR) and Methionine synthase reductase (MTRR) also are highly correlated with MTAP, CDKN2A, CDKN2B and other folate pathway genes. Besides the genetic alteration and the expression correlation analysis we also reviewed the previously published literature and found that MTAP is linked to the folate metabolism pathway through methionine cycle and the methionine salvage pathway^27,28^ (Fig. 1D). Moreover, MTAP loss is shown as a potential therapeutic target in chordoma^29^. In vitro and in vivo preclinical data using a urothelial carcinoma cell lines demonstrated increased sensitivity to pemetrexed by inducing DNA damage, and distorting nucleotide pools. In addition, it was found that an MTAP-knockdown increases sensitivity to pemetrexed. Therefore, tumor MTAP deficiency creates a metabolic vulnerability for therapy with antifolate agents such as pemetrexed^30^. As MTAP and TYMS are involved in important cellular processes, inhibition or loss of these genes have a major impact on the cellular processes and can possibly be explored further for use as therapeutic targets in chordoma.

### Preclinical activity of pemetrexed in chordoma mouse model

**Figure 2:**
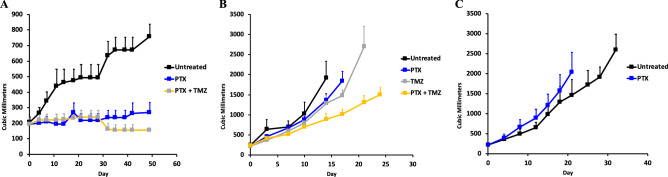
In vivo efficacy of pemetrexed in chordoma xenograft models. (**A**) Pemetrexed (PTX) and Temozolomide (TMZ) in U-CH1 CDX model (N = 4 in treatment groups, N = 3 in untreated control group); PTX *p* = 0.0003; PTX/TMZ *p* < 0.0001 (**B**). Pemetrexed, Temozolomide, and Pemetrexed/Temozolomide combination in the CF365 PDX model (N = 4 in treatment groups, N = 7 in untreated control group) (**C**). Pemetrexed in the SF10792 PDX model (N = 4 in treatment groups, N = 4 in untreated control group).

We tested the activity of pemetrexed (PTX) and temozolomide (TMZ) (a DNA alkylating chemotherapy) in mouse models using human chordoma patient derived xenograft (PDX) models established by the Chordoma Foundation. Of the three xenograft models studied, two of the three (U-CH1 and CF365) were tested with PTX and PTX with TMZ while the SF10792 was only tested with PTX. Pemetrexed was given at a dose of 100 mg/kg and TMZ was given at a dose of 25 mg/kg. Table S1 shows the establishment of three chordoma xenografts used in the in vivo drug testing. MTAP was found to be positive in the U-CH1 and CF365 xenograft models, while p16 was found to be positive in the U-CH1 and SF10792 xenograft models (Fig. S1). Using the U-CH1 cell-line derived xenograft (CDX) model, tumor volumes were significantly less (*p* < 0.05) in groups treated with pemetrexed (*p* = 0.0003), and pemetrexed/TMZ (*p* < 0.0001) compared to controls (269, 156 vs 759mm^3^, respectively after 49 days of treatment) (Fig. 2A). In 2 other PDXs (CF365, SF10792) there was not a significant effect of pemetrexed treatment (Fig. 2B,C).

### Clinical activity of pemetrexed in three chordoma patients

**Figure 3:**
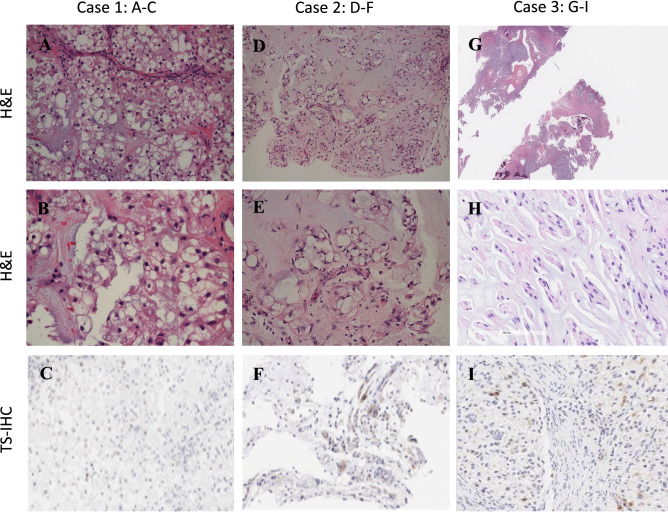
Microphotograph of brain biopsies. (**A**): (H&E, × 100). Metastatic chordoma involving cerebellum showing classic biphasic histology of vacuolated epithelioid cells in a myxoid matrix. (**B**): (H&E × 200). The epithelioid notochordal cells in this case have small, uniform nuclei. Multivacuolated (physaliphorous cells) are the hallmark of chordoma. (**C**): TS-IHC demonstrating no (0%) cytoplasmic immunostaining. (**D**) (H&E, × 100). Metastatic chordoma to chest wall showing similar classic histology with more prominent stromal component. (**E**) (H&E, × 200). The epithelioid cells with single large vacuoles may resemble signet ring cells. (**F**): TS-IHC demonstrating weak (1 +) cytoplasmic staining of < 10% of tumor cells. (**G**): (H&E, × 50). Metastatic chordoma involving skull base. (**H**): (H&E, × 200). The epithelioid cells with large vacuoles. (**I**): TS-ICH demonstrating weak (1 +) cytoplasmic staining of > 16% of tumor cells.

We treated three patients with recurrent chordoma with premetrexed after failing several lines of standard therapy and off-label therapies. In 2 responders, molecular profiling showed that TS was not expressed or low level (< 10%) in tumor and for the 1 non-responder, TS was expressed at higher level (> 10%). On pathology review, the three metastatic chordoma cases show classic chordomas with sheets of tumor cells arrayed in and indistinct cell boundaries (Fig. 3A,B, D–E, G,H). TS expression by IHC was performed from the paraffin blocks all cases (Fig. 3C,F,I). TS was found to be negative in the first two cases. Case 3 shows a low-moderate positive (> 16% cells with + 1 staining) TS expression in cytoplasm of tumor cells (Fig. 3I). Tumor molecular profiles from all cases are shown on Table 2.

**Table 2:** Comprehensive tumor profiling to assess DNA, RNA, and proteins in the three recurrent chordoma cases.

Biomarker	Method	Case 1	Case 2	Case 3
TML	NGS	Low 5 mutations/Mb	Intermediate 9 mutations/Mb	Low 1.66 mutations/Mb
MSI	NGS	Stable	Stable	N/A
CDKN2A	NGS	Amplification and mutation not detected	Amplification and mutation not detected	N/A
MGMT	PyroSeq	Unmethylated	Unmethylated	N/A
MLH1	IHC	Positive	Positive	N/A
MSH2	IHC	Positive	Positive	N/A
MSH6	IHC	Positive	Positive	N/A
PD-1	IHC	Positive	Negative	N/A
PMS2	IHC	Positive	Positive	N/A
PD-L1	IHC	Negative	Negative	N/A
TS	IHC	Negative Tumor Stained: 10% Intensity: 1 +	Negative Tumor Stained: 0% Intensity: 0 +	Positive Tumor Stained: > 16% Intensity: 1 +

*TML* tumor mutational load, *MSI* microsatellite instability, *CDKN2A* Cyclin dependent kinase inhibitor 2A, *MGMT* O6-methylguanine (O6-MeG)-DNA methyltransferase, *MLH1* MutL homolog 1, *MSH2* MutS homolog 2, *MSH6* MutS homolog 6, *PD-1* Programmed cell death protein 1, *PMS2* PMS1 homolog 2, *PD-L1* Programmed death-1 ligand 1, *TS* Thymidylate Synthase.

**Figure 4:**
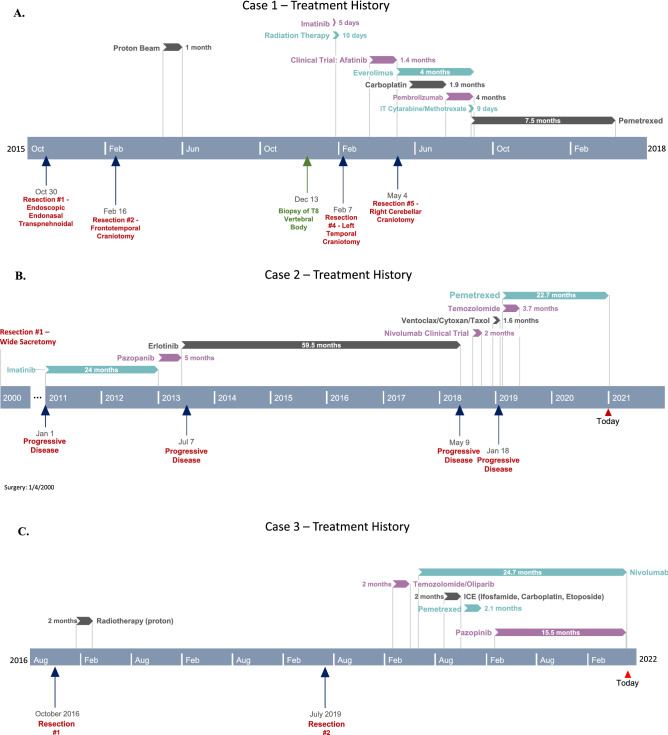
Case 1, 2, and 3 Treatment History. (**A**) Case 1 Treatment history course including all prior resections, standard therapy, and off-label treatments. (**B**) Case 2 Treatment history including prior resection, standard therapy, and off-label treatments. (**C**) Case 3 Treatment history including prior resection, standard therapy, and off-label treatments.

**Figure 5:**
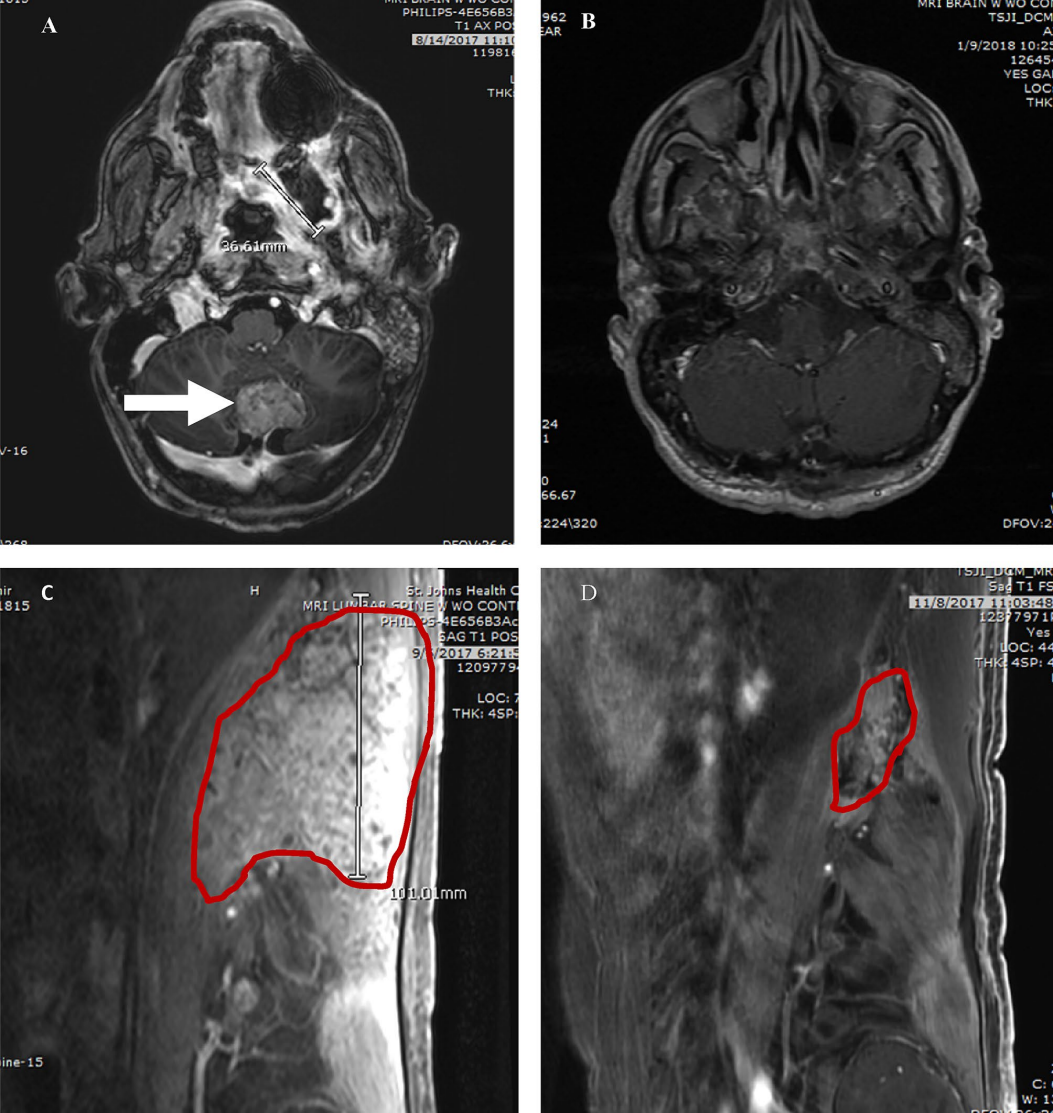
Case 1 MRI Brain and Retroperitoneum. Post-contrast MRI of patient with metastatic chordoma in brain (**A**) and retroperitoneum (**C**) before starting pemetrexed 900 mg/m^2^ and 5 months post-treatment in brain (**B**) and in 2 months retroperitoneum (**D**).

Case 1 is a 56-year-old right-handed male diagnosed with clival chordoma in October 2015. The patient had undergone surgery to obtain a wide excision the same month, followed by a re-excision in February 2016 and adjuvant proton therapy in April 2016. In November 2016 he had recurrence in the palate and was treated with radiotherapy and imatinib (best response stable disease). In December 2016 metastatic disease was found in the brain, dura, leptomeninges, bone, and lung. Figure 4A shows his course of treatment history including all prior resections, standard therapy, and off-label treatments (best response stable disease). CARIS profile showed TS negative by IHC and the patient started pemetrexed at 900 mg/m^2^ for 4 cycles. Serial magnetic resonance imaging (MRI) was done to monitor treatment response. There were partial responses in the brain and body initially as seen on MRI (Fig. 5). He continued to improve with complete response in the brain and almost complete responses in all measurable lesions in the body and leptomeninges. Despite the dramatic imaging response, patient in Case 1 passed away 6 months later after stopping treatment due to poor functional status from multiple other medical issues.

**Figure 6:**
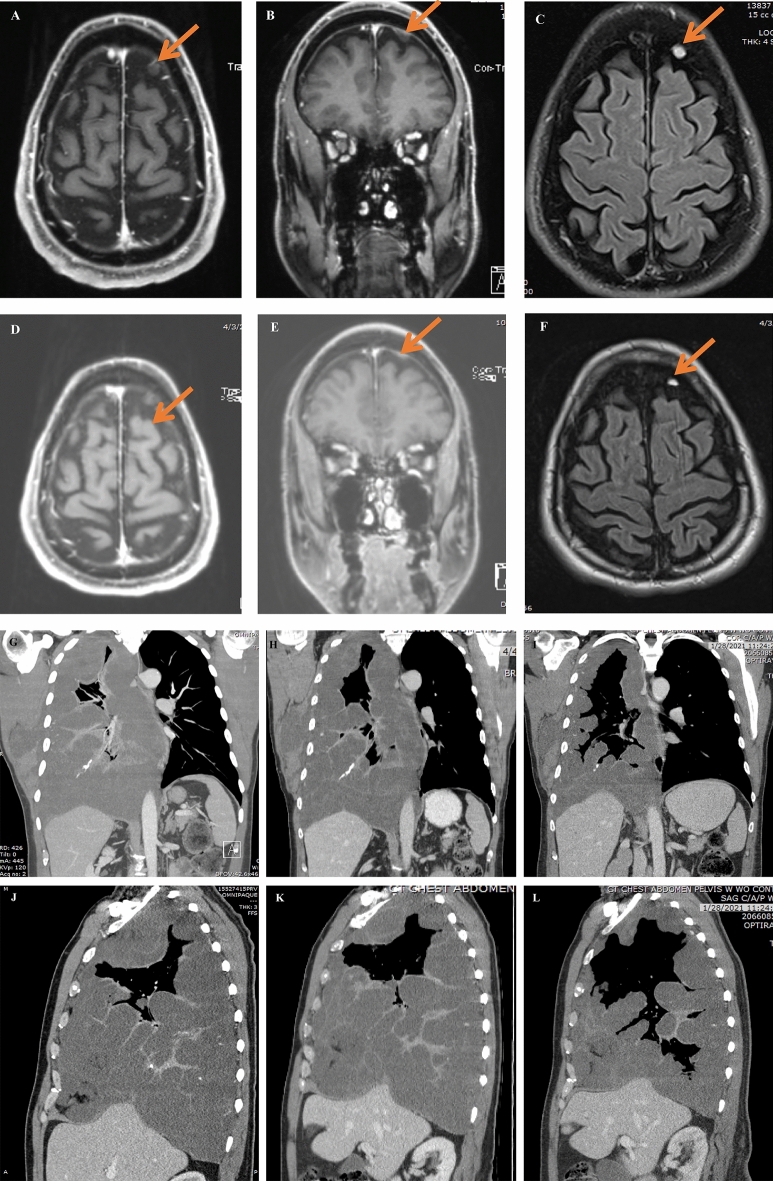
Case 2 MRI Brain and MRI Chest. MRI Brain: Post-contrast MRI of patient with metastatic chordoma in dura of brain (**A**-axial, **B**-coronal) and on T2/FLAIR (**C**) showing the lesion before starting pemetrexed 900 mg/m^2^ and 6 weeks post-treatment in brain (**D**-axial, **E**-coronal) and on T2/FLAIR (**F**) showing response. CT Chest: Post-contrast Chest CT of patient with metastatic chordoma to lung and soft tissue of axilla (**G**-coronal, **J**-sagittal) showing the large lesion filling most of right lung before starting pemetrexed 900 mg/m^2^, 6 weeks post-treatment (**H**-coronal, **K**-sagittal) showing shrinkage of tumor, and 2 years later on pemetrexed showing continued shrinkage of tumor and expansion of air ways in right lung and reduced mass effect and shift of mediastinal contents (**I**-coronal, **L**-sagittal).

Case 2 is that of a 64-year-old right-handed male who was diagnosed with stage IV sacral chordoma. In January 2000 he underwent wide sacral resection with reconstruction followed by serial imaging. In 2011 he experienced new pelvic recurrence and bilateral lung metastases. His treatment history outlines various drugs which were used prior to starting pemetrexed (Fig. 4B). Despite treatment with various drugs (best responses were stable disease), he continued to have recurrent disease progression with brain metastasis. His CARIS profile showed TS negative by IHC. In February 2019 he started pemetrexed 900 mg/m^2^ every three weeks plus metronomic temozolomide 100 mg daily for only 4 cycles. He then continued Pemetrexed monotherapy, and to date, he remains on pemetrexed every 3 weeks and has been tolerating it well. Serial imaging showed continuous partial responses in brain and chest lesions (Fig. 6). He currently continues on pemetrexed 900 mg/m^2^ every 3 weeks having already received 28 cycles of treatment for 22 months. In both patients, the treatment of pemetrexed was tolerated well with reports of only mild intermittent nausea. The tumor measurements in both cases suggest a partial response as determined by RECIST criteria.

**Figure 7:**
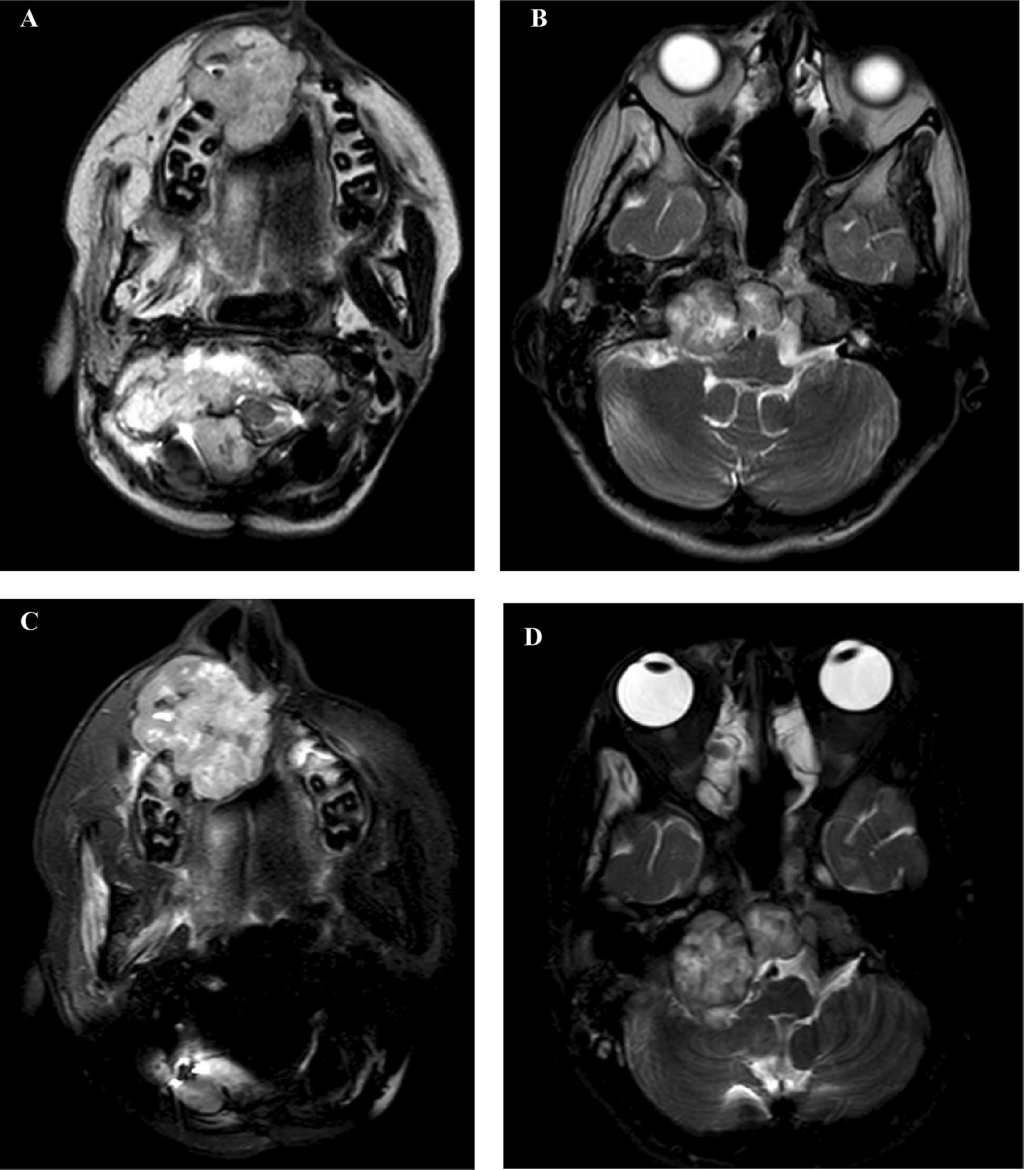
Case 3 MRI Brain. Axial T2 MRI sequences of patient with chordoma; (**A**,**B**) before starting pemetrexed 900 mg/m^2^. (**C**,**D**) after 3 cycles of pemetrexed with slow growth.

Case 3 is that of a 15-year-old male who was diagnosed with a clival cervical chordoma in October 2016 and underwent surgical resection followed by radiotherapy. He had radiographic recurrence and underwent a second subtotal resection in July 2019. Figure 4C shows his course of treatment history. He was given single agent pemetrexed in November 2020 and within 3 cycles there was significant tumor progression. MRI brain done pre- and post-pemetrexed treatment at three months showed a slowed tumor growth response to this drug (Fig. 7). Pemetrexed was discontinued and he was started on an alternative treatment.

## Discussion

Local recurrence rates in chordoma ranges from 43–85% with a 5- and 10-year overall survival rate of 70 and 40% respectively, leading to a poor quality of life^31,32^. To date, no effective treatments exist, and repeat resection or radiation are most used for such patients, but both carry a significant risk for morbidity and mortality. Systemic chemotherapy has been tried without clear success^33^. Here, we showed the activity of pemetrexed in preclinical human chordoma xenograft models and in two cases of relapsed chordoma treated off label. As seen on the MRIs, objective responses were observed. Such responses have not been previously documented with systemic chemotherapy^34^, with the exception of anecdotal case reports^35^. These findings suggest a possible systemic therapy option for the treatment of recurrent chordoma.

Tumors from two of the three cases treated with off-label pemetrexed were TS negative by IHC, which may suggest a possible mechanism for the antifolate activity of pemetrexed to be effective in this disease type. However, the sample size is too small to decide on whether TS expression correlated with response in these limited preclinical models which we know are not fully representative of the in vivo biology especially we selective for faster growing cell in vitro. Pemetrexed has been used widely in non-small cell lung cancer and gastrointestinal cancer with variable treatment response observed with different pathological types of tumors^36^.

Pemetrexed has been approved by the Food and Drug Administration (FDA) to treat locally advanced or metastatic non-squamous non-small cell lung cancer and malignant mesothelioma. Methotrexate has been used for decades to treat a variety of CNS malignancies, though the administration of high-dose methotrexate for gliomas has been used in limited practice with mixed success. Pemetrexed offers several potential advantages over methotrexate in the treatment of CNS cancers including higher affinity for folate transporters, superior polyglutamation kinetics, and broader anti-folate activity, and more favorable CSF penetration. Furthermore, its high solubility in water reduces the risk of renal failure and eliminates the need for urine alkalinization and aggressive pre-hydration which allows it to be given in the outpatient setting. Imatinib has been the most thoroughly evaluated systemic agent in chordoma, however it is not approved by the FDA for the treatment of chordoma. In a single-arm phase II study of Imatinib, radiographic response rate of only 1 of 50 patients (2%) was observed, despite stable disease in 35 of 50 patients (70%). The median progression free survival (PFS) was 9 months^37^. In a separate retrospective analysis, 17 of 17 patients having advanced disease and PDGFR expression were stable, but no radiographic responses were observed^38^.

Recent activity in the treatment of primary or secondary CNS malignancies was demonstrated with pemetrexed. In a study of relapsed or refractory primary CNS lymphoma (PCNSL), 11 patients were treated with pemetrexed 900 mg/m^2^ given every 3 weeks with low-dose dexamethasone, folate, and B12 supplementation. With 10 of those patients having failed prior high-dose methotrexate, 4 patients demonstrated complete response, 2 patients had partial response, and 4 patients had stable disease. The most common adverse events were thrombocytopenia, leukopenia, anemia, fatigue, ALT/AST elevation, and infection; and the most severe was pneumonia combined in one patient with neutropenia^39^.

Warwick et al. reported on a phase 2 trial of pemetrexed in children and adolescents with refractory solid tumors including CNS tumors^40^. Pemetrexed was administered to 72 patients at a dose of 1,910 mg/m^2^ as an intravenous infusion every 21 days along with dexamethasone, vitamin B12, and daily multivitamin supplementation. Although this regimen did not show evidence of objective anti-tumor activity in the childhood tumors studied, there were 5 patients with stable disease and the regimen was tolerable. The most common toxicities related to study treatment were neutropenia (44%), anemia (35%), elevated alanine transaminase (35%), and thrombocytopenia (30%).

A phase II study of pemetrexed was reported by Kumthekar et al. in patients with brain metastases or leptomeningeal metastases along with results of a second study assessing pemetrexed levels in cerebrospinal fluid (CSF)^41^. Twenty-one patients were treated with pemetrexed at doses of 500 mg/m^2^ (n = 3), 750 mg/m^2^ (n = 3), 900 mg/m^2^ (n = 12), or 1,050 mg/m^2^ (n = 3) with dexamethasone, folate, and B12 supplementation. The most common toxicities were myelosuppression, elevated transaminases, nausea/vomiting, and fatigue. Best radiographic response was 1 partial response, 10 stable disease, and 10 progressive disease, with no difference noted based on dose of pemetrexed. Pemetrexed was measurable in CSF (n = 3) with a concentration of < 5% of plasma drug concentration. Table S2 shows results of systemic therapies used in chordoma from clinical trials, case series, and case studies.

In a 40-patient study of recurrent gliomas and brain metastases, 65% of patients were found to have TS negative expression, and 10 of these TS negative patients were treated with pemetrexed. Out of those 10 patients, there was 1 partial response and 8 patients with stable disease. Toxicities were very mild except for 2 patients experiencing grade 3–4 adverse events who also received cisplatin. Thus, pemetrexed appears to have favorable efficacy in treating TS negative primary and systemic malignancies with CNS metastases^42^.

Pemetrexed appears to have a favorable efficacy for the treatment of TS negative secondary malignancies with brain metastases and in non-Glioblastoma primary tumors. Therefore, pemetrexed could be an excellent treatment option for those whose CNS malignancy is TS negative. The immunohistochemical evaluation of TS may be useful in selecting patients in future. A negative TS expression predicts therapeutic sensitivity of pemetrexed, suggesting it may be a potential target in the treatment for chordoma. Continued exploration of this agent either alone or in combination for chordoma is warranted and a clinical trial investigating the single agent pemetrexed is currently underway (NCT03955042).

## Materials and methods

### Patients

The three patients provided informed consent for Study JWCI-17–0401: Neurological Outcomes in Health and Disease which allows for the collection of clinical data and biological specimens from patients with tumors and neurological disorders as part of their routine medical evaluations in order to perform research studies that could advance patient care. The consent also allow for the publishing of identifying information and images from each participant. The study was approved by the Providence St. Joseph Health Institutional Review Board and all research was performed in accordance with relevant guidelines/regulations and in accordance with the Declaration of Helsinki.

### Animals and cells

The study was approved by XenoSTART (South Texas Accelerated Research Therapeutics) International Animal Care and Use Committee (IACUC). These preclinical studies conducted through the Chordoma Foundation’s Drug Screening Program at XenoSTART include protocol 09–001, which involves the development of new cancer models and anticancer drugs with the purpose of improving quality of life and survival for patients with cancer, and protocol 10–001, which is the Animal Health Surveillance (sentinel) program. With the use of experimental animals, the reporting in the manuscript follows the recommendations in the ARRIVE guidelines. Female athymic nude mice were purchased from The Jackson Laboratory for U-CH1 and NGS mice aged 8–12 weeks were purchased from Charles River Laboratories for SF10792 and CF365. Animals were housed under conventional conditions at the START facility and were cared for in accordance with institutional rules, state and federal laws, and ethical guidelines for experimental animal care. U-CH1 was obtained from the American Type Culture collection (ATCC) and was maintained in RPMI-1640 medium (Sigma Aldrich) with 10% FBS, 1-glutamine, penicillin, and streptomycin at 37 °C in 5% CO_2_. CF365 and SF10792 are patient-derived xenografts (PDX) carried in mice and generated as previously described and obtained from the Chordoma Foundation^43^.

The in vivo anti-tumor efficacy of pemetrexed and/or temozolomide was assessed in the tumor bearing models. Each mouse model was divided into different treatment groups (control-untreated, pemetrexed, and/or pemetrexed with temozolomide). Six to twelve weeks old, female athymic nude mice (for U-CH1 xenograft) or NSG mice (for CF365 and SF10792 xenografts) were implanted subcutaneously into the flank with tumor fragments from host animals. At predetermined time intervals, the tumor volume (TV) was measured (cubic millimeters). When tumors reached approximately 150–250 mm^3^, animals matched by tumor volume (TV) were randomized to control and treatment groups, each group containing 4–5 animals. Pemetrexed formulated in 0.9% NaCl was dosed at 100 mg/kg intraperitoneal (IP) on a schedule of 3 days on and 4 days off until the end of the study. Temozolomide was formulated in 0.5% carboxymethylcellulose and dosed at 25 mg/kg IP on a schedule of 5 days on and 2 days off to study end. The day of dosing was considered day 0. Animals were observed every day and weight was measured twice a week. Animal weight data and TV were electronically collected with a scale and digital caliper, respectively. Tumor dimensions were converted to TV using the formula: TV (mm^3^) = width (mm^2^) x length (mm) × 0.52. Animals reaching a mean TV of > 2.5 cm^3^ were removed from the study. The study endpoint was when the untreated group’s mean TV reached 2 cm^3^ or 28 days (for U-CH1), 42 days (for SF10792), or 1.5 cm^3^ (for CF365), whichever came first. Percent tumor growth inhibition was determined for treatment versus control groups using initial and final mean TV measurements. Statistical analysis was performed using a two-way ANOVA followed by the Dunnett multiple comparisons test.

### Tumor specimens

Routine hematoxylin and eosin staining were performed on paraffin sections from archival tissue blocks of the three patients for assessing tumor type and degree of cellular differentiation.

### Thymidylate synthase immunohistochemistry

TS expression in archival tumor tissues for the three patient cases was evaluated by Neogenomics for high-definition IHC staining using a polymer technology-based system. TS was stained via automated IHC instrument on the Leica Bond III (Lecia Biosystems, Melbourne, Australia). Staining intensity was assessed using the scale 0: no staining, 1: weak staining, 2: moderate staining, and 3: intense staining.

### Molecular profile

Molecular profiling of both cases by submitted to Caris Life Sciences using Next Gen Sequencing (NGS, NextSeq, Illumina, on a panel of 592 genes) and IHC. Tumor mutational load was calculated by counting nonsynonymous missense mutations in the 592 genes sequenced (1.4 megabase) and microsatellite instability (MSI) was measured by direct analysis of known MSI loci in the target regions of the sequenced genes.

### Bioinformatics analysis

Bioinformatics analysis of Chordoma datasets in terms of alteration, expression and network analysis.

#### Genetic alteration

Specific genes of interest including MTAP, CDKN2A, CDKN2B, CDKN2C and folate pathway genes were queried in the Personalized oncogenomics cBioPortal (https://www.personalizedoncogenomics.org/cbioportal/study?id=pog_chdm#summary). An oncoprint containing the genetic alteration (deep deletion) frequency was obtained for the 15 queried genes in three chordoma samples.

#### Network

Protein–protein interaction network was obtained using the STRING database version 11.0 (https://string-db.org). The same list of 15 genes as used for determining the genetic alteration in cBioPortal were employed as input in the STRING database to obtain the interaction network with these settings—full STRING network for the network type, minimum required interaction score of 0.7 and high FDR stringency of 1%. Kmeans clustering was used to cluster the network with a specified input of two clusters.

#### Expression analysis

Chordoma samples were obtained from the Array Express dataset E-MEXP-353 (https://www.ebi.ac.uk/arrayexpress/experiments/E-MEXP-353/)^44^. The raw CEL files of the Affymetrix array were downloaded and analyzed using the “maEndToEnd” package in R. Background correction, normalization and expression calculation was carried out for the four chordoma samples using the “oligo::rma” function in R. The Affymetrix probes were annotated to genes using the “hgu133a.db” annotation file. For genes with multiple probes (two or more), the probe id with the primary transcript variant was taken into consideration for further analysis. The primary transcript variant was obtained using the Gencode GRCh37 (release 38) GTF annotation file. Expression of the specific genes of interest were obtained and then correlation of the gene expression was plotted using the “corrplot” package in R with hierarchical clustering. R version 4.1.0^45^ was used for analyses and generating plots.

### Ethics statement

Authors have obtained informed consent from the patients for the inclusion of their medical and treatment history within this report.

## Supplementary Information

Supplementary Figure S1.

Supplementary Table S1.

Supplementary Table S2.

## Data Availability

Data is from clinical care of patients and via collaboration with CARIS Life Sciences. The datasets used and/or analyzed during the current study available from the corresponding author on reasonable request.
