# Supplementary material for: Activity of pemetrexed in pre-clinical chordoma models and humans
Source: Sci Rep. 2023 May 5;13:7317. doi: 10.1038/s41598-023-34404-4 (PMC10163028; doi:10.1038/s41598-023-34404-4)
Supplement: Supplementary file 1 — Supplementary Figure S1. [file 41598_2023_34404_MOESM1_ESM.pdf]

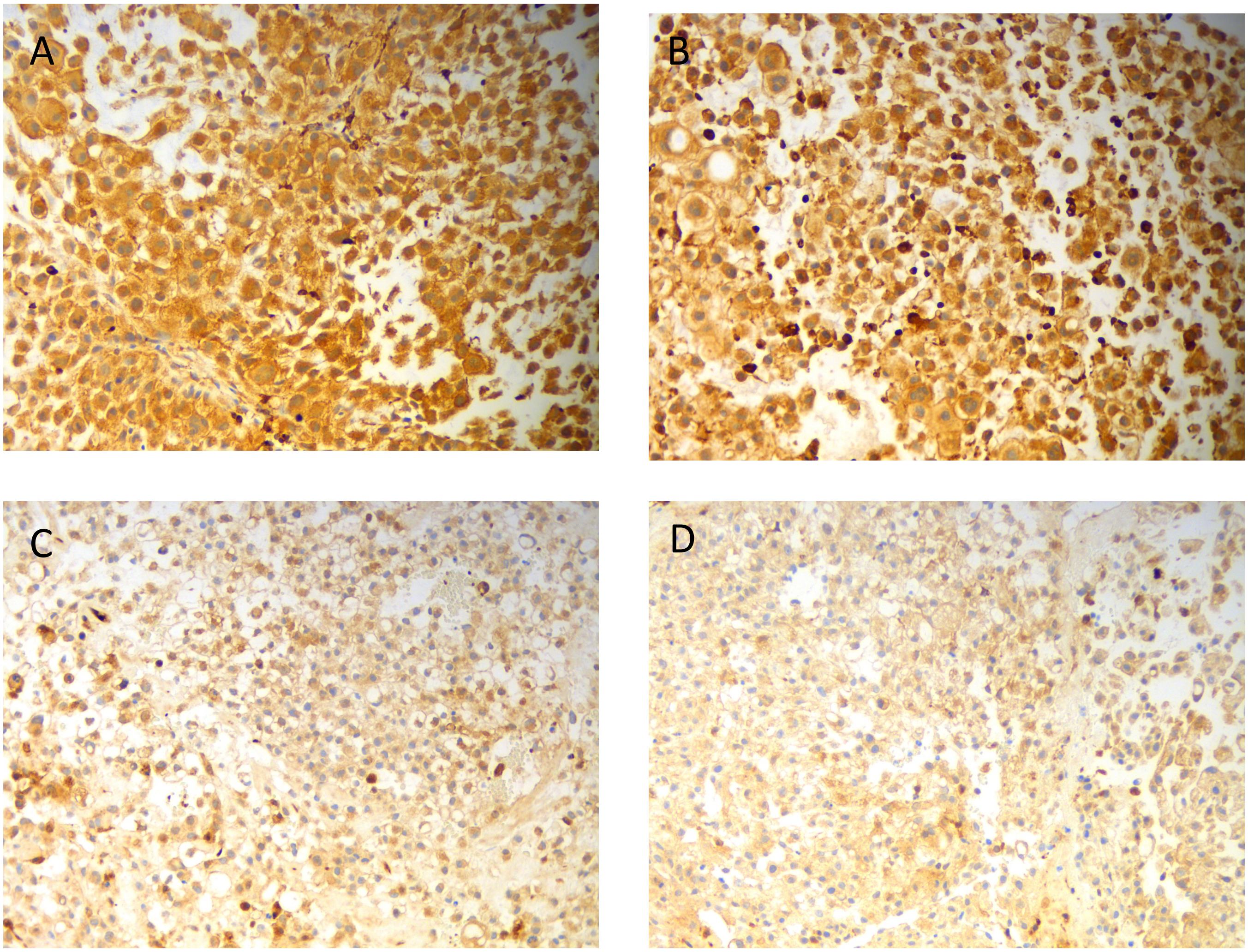

Fig. S1. Immunohistochemical staining of U-CH1 p16 and SF10792 MTAP
(A,B). U-CH1 cell line shows strong and diffuse nuclear and cytoplasmic p16 immunoreactivity (score, 2) **(C,D).** SF10792 cell line shows weak methylthioadenosine phosphorylase (MTAP) staining (score 0,1)
