# Supplementary material for: Activity of pemetrexed in pre-clinical chordoma models and humans
Source: Sci Rep. 2023 May 5;13:7317. doi: 10.1038/s41598-023-34404-4 (PMC10163028; doi:10.1038/s41598-023-34404-4)
Supplement: Supplementary file 2 — Supplementary Table S1. [file 41598_2023_34404_MOESM2_ESM.docx]

**Table. S1**. **Establishment of three chordoma xenograft models**

| **Model Name** | **Model Type** | **Tumor Location** | **Disease Status** | **Adult (>19)/ Pediatric** | **Exposure to Pemetrexed** | **Exposure to TMZ + Pemetrexed** | **p16 Intensity*** | **MTAP Intensity*** | **TS Intensity*** |
| --- | --- | --- | --- | --- | --- | --- | --- | --- | --- |
| **U-CH1** | CDX | Sacral | Recurrent | Adult | Y | Y | 2+, 2+ | 2+, 2+ | 2+, 2+ |
| **CF365** | PDX | Clival | Metastatic | Pediatric | Y | Y | 0, 0 | 1+, 1+ | 3+, 3+ |
| **SF10792** | PDX | Clival | Primary | Adult | Y | N | 2+, 2+ | 0, 1+ | 3+, 3+ |

Abbreviations: CDX: Cell-line derived xenograft; PDX: Patient derived xenograft; Y: Yes; N: No; TS: Thymidylate Synthase

* IHC Experiment repeated twice
