# Supplementary material for: Activity of pemetrexed in pre-clinical chordoma models and humans
Source: Sci Rep. 2023 May 5;13:7317. doi: 10.1038/s41598-023-34404-4 (PMC10163028; doi:10.1038/s41598-023-34404-4)
Supplement: Supplementary file 3 — Supplementary Table S2. [file 41598_2023_34404_MOESM3_ESM.pptx]

## Slide 1
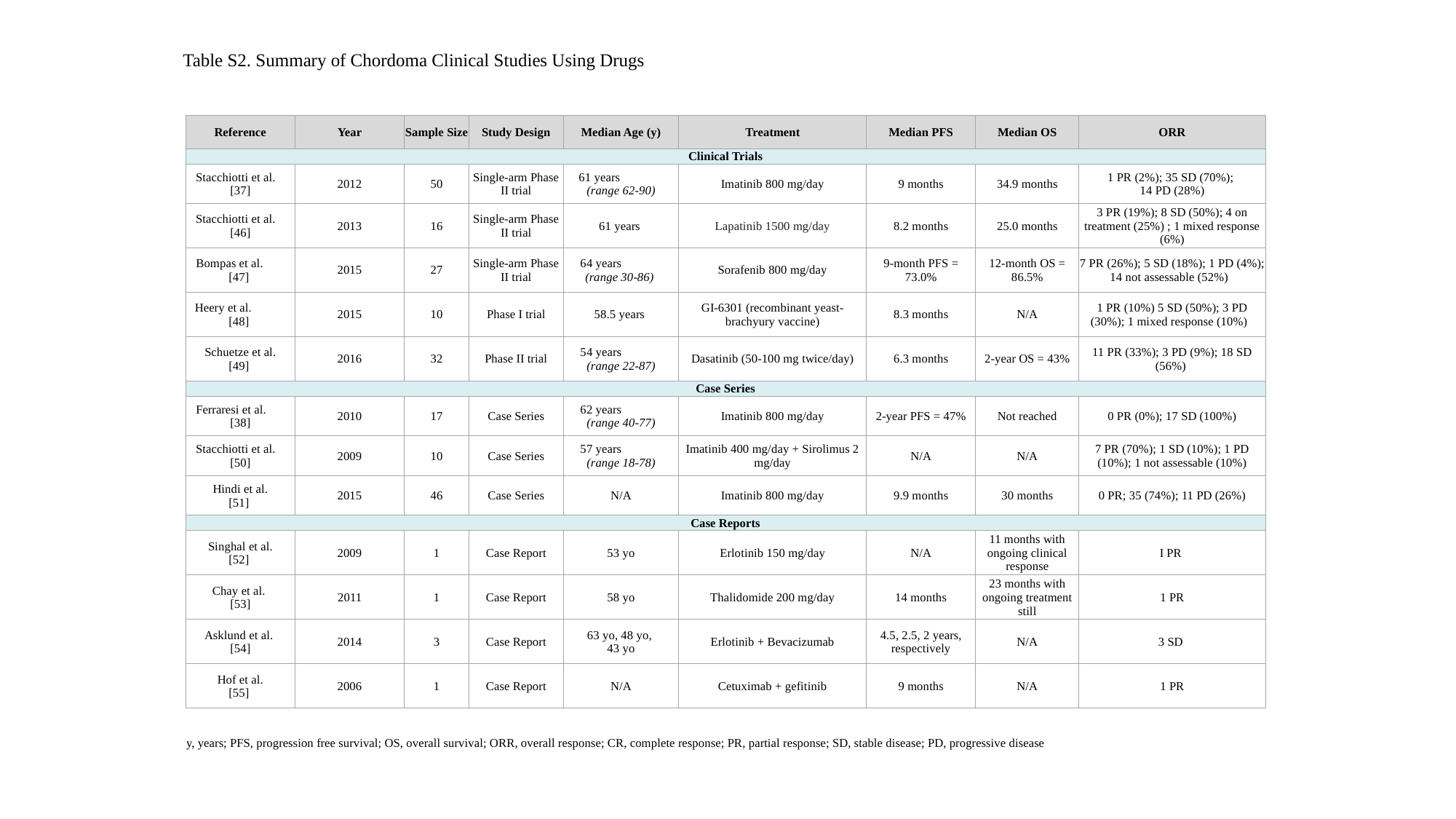

Table S2. Summary of Chordoma Clinical Studies Using Drugs
| Reference | Year | Sample Size | Study Design | Median Age (y) | Treatment | Median PFS | Median OS | ORR |
| --- | --- | --- | --- | --- | --- | --- | --- | --- |
| Clinical Trials | | | | | | | | |
| Stacchiotti et al. [37] | 2012 | 50 | Single-arm Phase II trial | 61 years (range 62-90) | Imatinib 800 mg/day | 9 months | 34.9 months | 1 PR (2%); 35 SD (70%); 14 PD (28%) |
| Stacchiotti et al. [46] | 2013 | 16 | Single-arm Phase II trial | 61 years | Lapatinib 1500 mg/day | 8.2 months | 25.0 months | 3 PR (19%); 8 SD (50%); 4 on treatment (25%) ; 1 mixed response (6%) |
| Bompas et al. [47] | 2015 | 27 | Single-arm Phase II trial | 64 years (range 30-86) | Sorafenib 800 mg/day | 9-month PFS = 73.0% | 12-month OS = 86.5% | 7 PR (26%); 5 SD (18%); 1 PD (4%); 14 not assessable (52%) |
| Heery et al. [48] | 2015 | 10 | Phase I trial | 58.5 years | GI-6301 (recombinant yeast-brachyury vaccine) | 8.3 months | N/A | 1 PR (10%) 5 SD (50%); 3 PD (30%); 1 mixed response (10%) |
| Schuetze et al. [49] | 2016 | 32 | Phase II trial | 54 years (range 22-87) | Dasatinib (50-100 mg twice/day) | 6.3 months | 2-year OS = 43% | 11 PR (33%); 3 PD (9%); 18 SD (56%) |
| Case Series | | | | | | | | |
| Ferraresi et al. [38] | 2010 | 17 | Case Series | 62 years (range 40-77) | Imatinib 800 mg/day | 2-year PFS = 47% | Not reached | 0 PR (0%); 17 SD (100%) |
| Stacchiotti et al. [50] | 2009 | 10 | Case Series | 57 years (range 18-78) | Imatinib 400 mg/day + Sirolimus 2 mg/day | N/A | N/A | 7 PR (70%); 1 SD (10%); 1 PD (10%); 1 not assessable (10%) |
| Hindi et al. [51] | 2015 | 46 | Case Series | N/A | Imatinib 800 mg/day | 9.9 months | 30 months | 0 PR; 35 (74%); 11 PD (26%) |
| Case Reports | | | | | | | | |
| Singhal et al. [52] | 2009 | 1 | Case Report | 53 yo | Erlotinib 150 mg/day | N/A | 11 months with ongoing clinical response | I PR |
| Chay et al. [53] | 2011 | 1 | Case Report | 58 yo | Thalidomide 200 mg/day | 14 months | 23 months with ongoing treatment still | 1 PR |
| Asklund et al. [54] | 2014 | 3 | Case Report | 63 yo, 48 yo, 43 yo | Erlotinib + Bevacizumab | 4.5, 2.5, 2 years, respectively | N/A | 3 SD |
| Hof et al. [55] | 2006 | 1 | Case Report | N/A | Cetuximab + gefitinib | 9 months | N/A | 1 PR |
| | | | | | | | | |
| y, years; PFS, progression free survival; OS, overall survival; ORR, overall response; CR, complete response; PR, partial response; SD, stable disease; PD, progressive disease | | | | | | | | |
